# Supplementary material for: The ‘ForensOMICS’ approach for postmortem interval estimation from human bone by integrating metabolomics, lipidomics, and proteomics
Source: eLife. 2022 Dec 30;11:e83658. doi: 10.7554/eLife.83658 (PMC9803353; doi:10.7554/eLife.83658)
Supplement: Source code 1. [file elife-83658-code1.zip › 22-09-2022-RA-eLife-83658/Source Code File 1.pdf]

The "ForensOMICS" approach to forensic post-mortem interval estimation: combining metabolomics, lipidomics and proteomics for the analysis of human bone

Andrea Bonicelli

2022-11-05

Table of Contents

Univariate Statistic ... 2
Test Univariate Statistic ... 2
Plot Univariate Statistic ... 3
Multivariate Statistic - Per Block ... 5
Multivariate Statistic - Integration ... 7
Model Validation ... 12

```
# Load packages
library(readr)
library(mixOmics)
library(BiocParallel)
library(RVAideMemoire)
library(tidyr)
library(rstatix)
library(reshape)
library(ggpubr)

# Load data
pData <-
  read.csv("pdata.csv", row.names = 1)
Prot <-
  as.matrix(read.csv("Prot.csv", row.names = 1, check.names = FALSE))
Hilic <-
  as.matrix(read.csv("Hilic.csv", row.names = 1, check.names = FALSE))
Lip <-
  as.matrix(read.csv("Lip.csv", row.names = 1, check.names = FALSE))

PMI <- as.vector(pData$PMI)
Age <- as.vector(pData$Age)
Deposition <- as.vector(pData$Deposition)
Individual <- as.vector(pData$Individual)

TestList <- list(
```

```
  Metabolite = Hilic,
  Protein = Prot,
  Lipid = Lip,
  PMI = as.factor(PMI),
  Age = as.factor(Age),
  Deposition = as.factor(Deposition),
  Individual = as.factor(Individual)
)

data = list(
  Metabolite = TestList$Metabolite,
  Protein = TestList$Protein,
  Lipid = TestList$Lipid
)
```

Univariate Statistic

Test Univariate Statistic

Univariate statistics included non-parametric Kruskal-Wallis (KW) test for global p-value testing of the overall difference in the samples divided by PMI. Pairwise comparison, despite the small sample size, was carried out Dunn's test for comparing mean rank of the different PMIs and p-value was adjusted using Holm–Bonferroni method. This allows retention the dependent ranking that produced the KW test statistic.

```
# metabolomics block
Hilic_melted <- melt(Hilic)
Hilic_melted$SampleName <- rownames(Hilic)

Metab_KW <- Hilic_melted %>%
  group_by(variable) %>%
  kruskal_test(value ~ PMI) %>%
  add_significance()

Metab_Pair <- Hilic_melted %>%
  group_by(variable) %>%
  dunn_test(value ~ PMI,
            p.adjust.method = "holm") %>%
  add_significance()

write_csv(Lip_KW, "Lip_KW.csv")
write_csv(Lip_Pair, "Lip_Pair.csv")

# lipidomcis block
Lip_melted <- melt(Lip)
Lip_melted$SampleName <- rownames(Lip)

Lip_KW <- Lip_melted %>%
  group_by(variable) %>%
  kruskal_test(value ~ PMI) %>%
```

```
  add_significance()

Lip_Pair <- Lip_melted %>%
  group_by(variable) %>%
  dunn_test(value ~ PMI,
                      p.adjust.method = "holm") %>%
  add_significance()


write_csv(Lip_KW, "Lip_KW.csv")
write_csv(Lip_Pair, "Lip_Pair.csv")

# proteomics block
Prot_melted <- melt(Prot)
Prot_melted$SampleName <- rownames(Prot)

Prot_KW <- Prot_melted %>%
  group_by(variable) %>%
  kruskal_test(value ~ PMI) %>%
  add_significance()

Prot_Pair <- Prot_melted %>%
  group_by(variable) %>%
  dunn_test(value ~ PMI,
            p.adjust.method = "holm") %>%
  add_significance()

write_csv(Prot_KW, "Prot_KW.csv")
write_csv(Prot_Pair, "Prot_Pair.csv")
```

Plot Univariate Statistic

Plot can be roduced for supplementary materials not included in the manuscript. Please see univariate statistics table for reference.

```
# set same colors as the remaining analysis
my.colors = color.mixo(1:5)


# metabolomics block - box plot
hilic_box <- Hilic %>%

  dplyr::select(PMI, "α-Eleostearic acid":"Xanthine") %>%
  gather(Measure, Value, -PMI) %>%
  ggplot(aes(x = factor(PMI),
             y = Value,
             color = PMI)) +
  geom_boxplot(width = 0.4, lwd = .8)    +
  facet_wrap( ~ Measure, scales = "free_y") +
  theme_bw()  +
```

```
  scale_color_manual(values = my.colors) +
  rremove("xlab") + ylab("Relative intensity") +
  theme(axis.text.x = element_text(angle = 45, hjust=1))

ggsave(hilic_box, file="hilic_box.pdf", width=20, height=18)

# lipidomics block - box plot
lip_box <- Lip %>%

  dplyr::select(PMI, "Cer(d12:0_18:1)+HCOO":"Hex2Cer(d18:1_24:1)+HCOO") %>%
  gather(Measure, Value,-PMI) %>%
  ggplot(aes(x = factor(PMI),
             y = Value,
             color = PMI)) +
  geom_boxplot(width = 0.4, lwd = .8)   +
  facet_wrap(~ Measure, scales = "free_y") +
  theme_bw()  +
  scale_color_manual(values = my.colors) +
  rremove("xlab") + ylab("Relative intensity") +
  theme(axis.text.x = element_text(angle = 45, hjust=1))

ggsave(lip_box, file="lip_box.pdf", width=20, height=18)

lip1_box <- Lip %>%

  dplyr::select(PMI, "LPC(14:0)+HCOO":"PS(18:0_18:1)-H") %>%
  gather(Measure, Value,-PMI) %>%
  ggplot(aes(x = factor(PMI),
             y = Value,
             color = PMI)) +
  geom_boxplot(width = 0.4, lwd = .8)   +
  facet_wrap(~ Measure, scales = "free_y") +
  theme_bw()  +
  scale_color_manual(values = my.colors) +
  rremove("xlab") + ylab("Relative intensity") +
  theme(axis.text.x = element_text(angle = 45, hjust=1))

ggsave(lip1_box, file="lip1_box.pdf", width=20, height=16)

# lipidomics block - box plot
prot_box <- Prot %>%

  dplyr::select(PMI, "A1AT_HUMAN":"CERU_HUMAN") %>%
  gather(Measure, Value,-PMI) %>%
  ggplot(aes(x = factor(PMI),
             y = Value,
             color = PMI)) +
  geom_boxplot(width = 0.4, lwd = .8)   +
  facet_wrap(~ Measure, scales = "free_y") +
  theme_bw()  +
```

```
  scale_color_manual(values = my.colors) +
  rremove("xlab") + ylab("Relative intensity") +
  theme(axis.text.x = element_text(angle = 45, hjust=1))

ggsave(prot_box, file="prot_box.pdf", width=20, height=18)
```

Multivariate Statistic - Per Block

Multivariate statistics was performed by sparse partial least squared discriminant analysis (PLS-DA) for each omics assay. We aimed to maintain the same approach used for omics integration. Initial model were created without cross validation for each block (metab.plsda, lip.plsda, prot.plsda).

```
# create list
PMI <- as.vector(pData$PMI)
Age <- as.vector(pData$Age)
Deposition <- as.vector(pData$Deposition)
Individual <- as.vector(pData$Individual)

TestList <- list(
  Metabolite = Hilic,
  Protein = Prot,
  Lipid = Lip,
  PMI = as.factor(PMI),
  Age = as.factor(Age),
  Deposition = as.factor(Deposition),
  Individual = as.factor(Individual)
)

data = list(
  Metabolite = TestList$Metabolite,
  Protein = TestList$Protein,
  Lipid = TestList$Lipid
)

lapply(data, dim)

# metabolite plsda
metab.plsda <- splsda(TestList$Metabolite,
                      TestList$PMI,
                      ncomp = 10)


plotIndiv(metab.plsda,
  comp = c(1, 2),
  abline = TRUE ,
  ind.names = FALSE,
  legend = TRUE,
  title = 'PLS-DA Lipid',
  pch = as.factor(pData$Deposition),
```

```r
  legend.title.pch = 'Deposition',
  legend.title = 'PMI'
)

legend = list(
  legend = levels(TestList$PMI),
  col = unique(color.mixo(TestList$PMI)),
  title = "PMI",
  cex = 0.7
)

cim(
  metab.plsda,
  row.sideColors = color.mixo(TestList$PMI),
  keysize.label = 1,
  legend = legend,
  transpose = TRUE,
  margin = c(8, 15)
)

# lipid plsda
lip.plsda <- splsda(TestList$Lipid, TestList$PMI, ncomp = 10)

plotIndiv(
  lip.plsda,
  comp = c(1, 2),
  abline = TRUE ,
  ind.names = FALSE,
  legend = TRUE,
  title = 'PLS-DA Lipid',
  pch = as.factor(pData$Deposition),
  legend.title.pch = 'Deposition',
  legend.title = 'PMI'
)

cim(
  lip.plsda,
  row.sideColors = color.mixo(TestList$PMI),
  keysize.label = 1,
  legend = legend,
  transpose = TRUE,
  margin = c(8, 15)
)

prot.plsda <- splsda(TestList$Protein, TestList$PMI, ncomp = 5)

plotIndiv(
  prot.plsda,
  comp = c(1, 3),
  abline = TRUE ,
```

```r
  ind.names = FALSE,
  legend = TRUE,
  title = 'PLS-DA Lipid',
  pch = as.factor(pData$Deposition),
  legend.title.pch = 'Deposition',
  legend.title = 'PMI'
)

cim(
  prot.plsda,
  row.sideColors = color.mixo(TestList$PMI),
  keysize.label = 1,
  legend = legend,
  transpose = TRUE,
  margin = c(8, 15)
)
```

Multivariate Statistic - Integration

First step in the integration of the three omics assay was test correlation pairwise using spls().

```r
# generate three pairwise PLS models for blocks correlation
pls1 <- spls(TestList[["Metabolite"]], TestList[["Lipid"]])
pls2 <- spls(TestList[["Metabolite"]], TestList[["Protein"]])
pls3 <- spls(TestList[["Lipid"]], TestList[["Protein"]])

cor(pls1$variates$X, pls1$variates$Y)
cor(pls2$variates$X, pls2$variates$Y)
cor(pls3$variates$X, pls3$variates$Y)
```

Considering the moderate/high correlation between the blocks' features the square matrix was set to 0.1 will be used to prioritise the discriminating ability of the model.

```r
# for square matrix filled with 0.1s
design = matrix(
  0.1,
  ncol = length(data),
  nrow = length(data),
  dimnames = list(names(data),
                  names(data))
)

diag(design) = 0 # set diagonal to 0s

design
```

A preliminary model (basic.diablo.model) was created block.splsda() without variable selection of component tuning.

```r
# fit basic DIABLO model
basic.diablo.model <- block.splsda(
  X = data,
  Y = TestList$PMI,
  design = design,
  ncomp = 5,
  scale = TRUE
)

plotIndiv(
  basic.diablo.model,
  comp = c(1, 2),
  abline = TRUE ,
  ind.names = FALSE,
  legend = TRUE,
  title = 'Full model Sample Plots',
  pch = as.factor(pData$Deposition),
  legend.title.pch = 'Deposition',
  legend.title = 'PMI',
)
```

Optimal component number was set by using cross validation using the perf() function with the following parameters:

1. validation = “Mfold”
2. folds = 3
3. nrepeat = 100
4. multilevel = TestList$Individual

For cross validation, all replicates of the same subject at the same time must be excluded during each run of cross-validation using a multilevel approach.

```r
# Assess performance and build final model
perf.diablo <- perf(
  basic.diablo.model,
  validation = 'Mfold',
  folds = 3,
  nrepeat = 100,
  cpu = 8,
  auc = TRUE,
  multilevel = TestList$Individual
)

# plot output of tuning dashed=overall.ER full=Overall.BER
plot(
  perf.diablo,
  sd = TRUE,
  dist = c("all"),
  measure = 'overall',
  weighted = TRUE
```

```
)

# Set up final DIABLO model
# set the optimal component number
ncomp <-
  perf.diablo$choice.ncomp$WeightedVote["Overall.BER", "centroids.dist"]
```

For variable selection the grid search can be seen in test.KeepX variable and and the tuning was performed base in the following parameters:

1. validation = “Mfold”
2. folds = 3
3. nrepeat = 100
4. multilevel = TestList$Individual

```
#run model tuning
tune.PMI <- tune.block.splsda(
  X = data,
  Y = pData$PMI,
  ncomp = ncomp,
  design = design,
  test.keepX = test.keepX,
  validation = 'Mfold',
  folds = 3,
  nrepeat = 100,
  scale = TRUE,
  dist = "centroids.dist",
  progressBar = TRUE,
  cpu = 8,
  multilevel = TestList$Individual
)

# extract the optimal values of features to retain
list.keepX <- tune.PMI$choice.keepX
list.keepX

# fit the optimised DIABLO model
final.diablo.model <- block.splsda(
  X = data,
  Y = pData$PMI,
  ncomp = 3,
  keepX = list.keepX,
  design = design,
)

selectVar(final.diablo.model)

# plot results
plot(tune.PMI, optimal = TRUE)
```

```r
pdf(file = "cim_final.pdf",
    width = 12,
    height = 8)

cimDiablo(
  final.diablo.model,
  comp = 1,
  color.blocks = c('red', 'green', 'blue'),
  legend.position = "right",
  scale = TRUE,
  center = TRUE,
  size.legend = 1.5,
  row.names = TRUE,
  col.names = TRUE,
  margin = c(8, 20),
  transpose = TRUE
)

dev.off()

plotIndiv(
  final.diablo.model,
  comp = c(1, 2),
  abline = TRUE ,
  ind.names = FALSE,
  legend = TRUE,
  title = 'Final model Sample Plots',
  pch = as.factor(pData$Deposition),
  legend.title.pch = 'Deposition context',
  legend.title = 'PMI',
  subtitle = c('Metabolite', 'Protein', 'Lipids'),
  blocks = c(1, 2, 3)
)

ggsave(file="Indiv_final.pdf", width=8, height=6)

plotArrow(
  final.diablo.model,
  abline = TRUE ,
  ind.names = FALSE,
  legend = TRUE,
  title = 'Final model Sample Plots',
  pch = as.factor(pData$Deposition),
  legend.title.pch = 'Deposition context',
  legend.title = 'PMI',
  subtitle = c('Metabolite', 'Lipid', 'Protein')
) +
  theme_bw()
```

```r
ggsave(file="arrow_final.pdf", width=6, height=4.5)

plotVar(
  final.diablo.model,
  comp=c(1:2),
  abline = TRUE ,
  cutoff = 0.5,
  var.names = c(TRUE, TRUE, TRUE),
  legend = TRUE,
  pch = c(15, 16, 17),
  cex = c(4, 4, 4),
  overlap = FALSE
)

ggsave(file="var_final.pdf", width=12, height=4)

pdf(file = "loadings_final.pdf",
    width = 12,
    height = 8)

plotLoadings(
  final.diablo.model,
  comp = 1,
  contrib = 'max',
  method = 'median',
  legend = FALSE,
  subtitle = c('Metabolite', 'Lipid', 'Protein')
)

dev.off()

plotDiablo(final.diablo.model, ncomp = 1)

# plot all selected markers per block
plotMarkers(
  final.diablo.model,
  block = c('Metabolite'),
  comp = 1,
  title = 'Standardised values - Metabolomics',
  boxplot.width = 0.8,
  violin = FALSE
) +
  theme_bw()+
  theme(axis.text.x = element_text(angle = 45, hjust=1))

plotMarkers(
  final.diablo.model,
  block = 'Lipid',
```

```
  comp = 1,
  title = 'Standardised values - Lipidomics',
  boxplot.width = 0.8,
  violin = FALSE
) +
  theme_bw()+
  theme(axis.text.x = element_text(angle = 45, hjust=1))

plotMarkers(
  final.diablo.model,
  block = 'Protein',
  comp = 1,
  title = 'Standardised values - Proteomics',
  boxplot.width = 0.8,
  violin = FALSE
) +
  theme_bw()+
  theme(axis.text.x = element_text(angle = 45, hjust=1))
```

Model Validation

Validation of the method was tested by randomization test via permutation test according to the following settings:

1. validation = "Mfold"
2. k = 3
3. nperm = 999

```
# estimate the classification error rate via cross validation
Diablo.cv <- DIABLO.cv(
  final.diablo.model,
  method = "centroids.dist",
  validation = "Mfold",
  k = 3,
  repet = 100,
  cpu = 8,
  multilevel = TestList$Individual
)

Diablo.cv

# significance of the discrimination classification error rate via
permutation
Diablo.Perm <- DIABLO.test(
  final.diablo.model,
  method = "centroids.dist",
  validation = "Mfold",
  k = 3,
  nperm = 999,
  cpu = 8,
```

```
  multilevel = TestList$Individual
)

Diablo.Perm
```
